# A Mixed Methods Investigation of the Experience of Poverty Among a Population of Low-Income Parenting Women

**DOI:** 10.1007/s10597-017-0093-z

**Published:** 2017-02-06

**Authors:** Brittany C. L. Lange, Ana Luísa B. T. Dáu, Joanne Goldblum, Janet Alfano, Megan V. Smith

**Affiliations:** 1 Department of Social Policy and Intervention, Oxford University, Barnett House, 32 Wellington Square, Oxford, OX1 2ER UK; 2 Child Study Center, Yale School of Medicine, 40 Temple Street, Suite #6B, New Haven, CT USA; 3 National Diaper Bank Network, 155 East Street, Suite 101, New Haven, CT USA; 4 The Diaper Bank, P.O. Box 9017, New Haven, CT USA; 5 Department of Psychiatry, Yale School of Medicine, New Haven, CT USA; 6 Department of Chronic Disease Epidemiology, Yale School of Public Health, New Haven, CT USA

**Keywords:** Needs assessment, Basic needs, Psychological stress, Poverty, Maternal mental health

## Abstract

This study sought to operationalize poverty in the context of parenting specific to a sample of low-income mothers; to examine how mothers describe sources of stress related to poverty; and to explore how these experiences affect mothers’ parenting practices. Mothers trained in research methods administered surveys to other mothers in community settings assessing parenting stressors, mental wellbeing, basic needs, and goals. Women reported difficulty obtaining basic needs. Qualitatively, women described financial hardship, housing, employment status, and transportation as sources of stress, which influenced their parenting practices. These findings connect a mother’s inability to meet her basic needs with parenting quality, and suggest that programs promoting early childhood development through building the capacity of parents must focus on basic needs and strategies to alleviate poverty. Healthcare providers may be able to glean specific terminology utilized by women when they inquire about basic needs and form partnerships with basic needs providers.

## Introduction

The experience of poverty represents a significant public health issue. As of 2013, approximately 30.6 million adults and 14.7 million children in the United States were living in poverty (DeNavas-Walt et al. 2014). Poverty disproportionately affects females (DeNavas-Walt et al. 2014; Entmacher et al. 2014), with 25.2 million females living in poverty in the United States as of 2013, compared to 20.1 million males (DeNavas-Walt et al. 2014). Women of certain racial/ethnic groups (Hispanic, Native American and black) and age groups (younger cohorts) are more likely to experience poverty (DeNavas-Walt et al. 2014; Entmacher et al. 2014).

The experience of poverty can affect women’s emotional wellbeing. Women are more likely than their male counterparts to report stress in their life caused by the economy or money (American Psychological Association). Furthermore, women who experience poverty, including economic hardship, are more likely to experience depression (Manuel et al. 2012). Depressive symptoms are also likely to be higher among certain subgroups of low-income women. For example, in a sample of African American, single mothers receiving welfare, 40% reported depressive symptoms severe enough to meet diagnostic criteria for clinical depression (Coiro 2001). This result has been duplicated in recent qualitative research indicating that difficulties with emotional wellbeing are often exacerbated in samples of low-income, minority women, as many do not have health insurance or report concern that their healthcare provider may hold discriminatory beliefs (Lazear et al. 2008).

Poverty’s pernicious impact on infants and children has been well documented (Brooks-Gunn and Duncan 1997). Disturbances in the emotional wellbeing of mothers, such as those caused by anxiety and depressive symptoms and disorders, are associated with an increase in infants born at lower birth weight, shorter gestation, and overall adverse effects on fetal/infant development, as compared to mothers without these symptoms (Schetter and Tanner 2012). Children living in poverty are also more likely to have mothers who are experiencing depressive symptoms or high degrees of perceived stress (Berger et al. 2009), and higher psychological distress among parents living in poverty is associated with poorer psychological outcomes, including internalizing and externalizing behaviors, among children (Robila and Krishnakumar 2006). Additionally, social capital, including trust and participation, is restricted in low-income families, which has been found to affect children’s outcomes, including their mental health and behavior (De Silva et al. 2005; Parcel and Menaghan 1993).

Currently, the United States determines whether an individual is in poverty using income thresholds, which take into account the size of families and the age of individuals within these families (United States Census Bureau 2014). Though the use of income as a standard for poverty determination is widespread, some researchers have examined material hardship in relation to poverty, which includes concerns such as food insufficiency and utility disconnection, and found these measures to be more targeted measures of both deprivation and health outcomes (Beverly 2001; Gershoff et al. 2007; Heflin and Iceland 2009). Given these emerging conceptualizations of poverty, and their importance to maternal and child health, there is a growing need to operationalize poverty in order to tailor interventions for families to actual reported needs of families living in poverty.

While previous research has focused on specific aspects of poverty pertaining to low-income women, such as marriage and emotional health (Coiro 2001; Edin 2000; Edin and Reed 2005; Gibson-Davis et al. 2005), these studies for the most part have not looked specifically at how poverty affects parenting practices, but rather note there is a main effect of income on overall quality of parenting and self-reported parental efficacy (Waldfogel et al. 2010). As such, this study aimed to (1) operationalize poverty specific to a sample of low-income mothers, (2) examine how mothers describe sources of stress related to poverty, and (3) explore how the experience of poverty affects women’s parenting practices.

## Methods

### Procedures

The New Haven Mental health Outreach for Mothers (MOMS) Partnership, a collaborative of agencies dedicated to improving the wellbeing of women and families, conducted a needs assessment with a sample of parenting women between January of 2011 and June of 2015. This needs assessment was structured into six sections: (1) About Yourself, (2) Housing, (3) Your Family, (4) Basic Needs, (5) Your Physical Health and Emotional Well Being, and (6) Motherhood and Personal Goals, which were designed to capture systematic information specific to low-income parenting women, including information about their financial situation and their current sources of stress. The needs assessment consisted of both open- and close-ended questions, allowing for both qualitative and quantitative data analysis to occur.

Women were recruited for this needs assessment by community mental health ambassadors (CMHAs) who were women who lived in the local community and were chosen for their familiarity with local neighborhoods. CMHAs underwent extensive mental health and community training. For information on training procedures, please see (Smith and Kruse-Austin 2015). CMHAs initiated recruitment activities in settings typically frequented by parenting or pregnant women. Additionally, systematic recruitment activities took place in a number of community locations, including schools, stores, and parks, in an effort to recruit individuals evenly in neighborhoods throughout the community. CMHAs used standardized language for recruitment activities.

Participants were eligible to participate in the study if they met the following criteria: (1) were female, (2) lived in the urban city where the study took place, (3) were at least 18 years old, (4) were pregnant, parenting or served as the primary caregiver for a child who was under the age of 18, (5) were able to provide written informed consent, and (6) spoke Spanish, English or Farsi.

Upon establishing eligibility, needs assessments were administered by a trained CMHA who had undergone at least 2.5 days of training and had conducted both practice and supervised surveys. All women completed the assessment in person, and when needed, research staff members were available to read questions or to help with their interpretation of questions.

### Ethics

Approval for this study was obtained from the Yale University Institutional Review Board (IRB) and from the IRBs of organizations involved with the MOMS Partnership. CMHAs obtained written informed consent from all study participants and participants were given the right to skip questions within the needs assessment. Women were compensated for their time with a $10 gift card.

Additionally, the authors of this manuscript hold no conflicts of interest related to this research and are responsible for all content in this manuscript.

### Data Analysis

#### Quantitative Data Analysis

Data were restricted to those women who had completed the survey between January of 2013 and the beginning of June of 2015 to ensure consistency of measures across survey versions. Univariate analyses were conducted on variables related to the demographic characteristics, clinical characteristics, and basic needs of parenting women. Several questions related to basic needs were specific to diaper use. As diaper use is not applicable to older children, a subset of data were taken for basic needs questions related to diaper use with parents that had children under 4 years of age. SAS 9.4 was utilized for all statistical analyses.

#### Qualitative Data Analysis

Qualitative data analysis centered on the question, “What are the current stressors in your life?” All answers to this question were compiled (n = 436), and a coder trained in qualitative coding methodology independently coded these responses using principles of thematic analysis (Braun and Clarke 2006). Specifically, inductive analysis was utilized for its more data-driven nature (Braun and Clarke 2006). First, the researcher read and re-read all responses to become familiar with the data (Braun and Clarke 2006). Responses were then coded into broad themes and thematic maps were created to visualize and make connections between themes (Braun and Clarke 2006). As coding progressed, responses were categorized into sub-themes, with all themes and sub-themes given a corresponding definition to ensure consistency in coding. Upon completion of initial coding, this researcher entered all data into ATLAS.ti, and a second researcher, also trained in qualitative coding, was given a subset of 20% of responses to code. Researchers compared their codes on these responses and had an initial agreement of 76.7%, with Cohen’s Kappa calculated to be 0.73, indicating good inter-coder reliability (Altman 1991). Upon completion of inter-coder reliability measures, remaining discrepancies were discussed and codes were revised as necessary until consensus was reached. Lastly, key words used by parenting women within each theme were identified by the primary coder. A search function assisted in the calculation of the frequencies of key words.

## Results

### Operationalizing the Experience of Poverty Among Parenting Women

Descriptive characteristics of the sample (N = 670) can be found in Table 1. The majority of the women in the study were between the ages of 25–44 (66.3%; n = 381). Self-reported African American (non-Hispanic) racial and ethnic background represented the largest race/ethnicity in the study (66.2%; n = 435), followed by Hispanics (10.5%; n = 69). The majority of the sample was born in the United States (92.3%; n = 609) and spoke English as the primary language at home (93.9%; n = 620). The majority of the sample had completed high school or less (53.7%; n = 344). Most women were unemployed (54.8%; n = 290), rented an apartment (58.7%; n = 379), and reported some level of feeling unsafe or fearful in their neighborhood (55.8%; n = 243). The majority of the women in the sample had insurance through Medicaid (80.4%; n = 448). Finally, 57.6% (n = 373) had CES-D scores of 16 or higher.

**Table 1:** Demographic and clinical characteristics of parenting women (N = 670)

Characteristic	n	%
Age group (n = 575)		
18–24	45	7.8
25–34	238	41.4
35–44	143	24.9
45–54	101	17.6
55+	48	8.3
Race/ethnicity (n = 657)		
White, non-Hispanic	54	8.2
Black or African American, non-Hispanic	435	66.2
White, Hispanic	69	10.5
Black or African American, Hispanic	60	9.1
Asian	5	0.8
Other	34	5.2
Born in the US (n = 660)		
Yes	609	92.3
No	51	7.7
Primary language spoken at home (n = 660)		
English	620	93.9
Spanish	34	5.2
Other	6	0.9
Highest level of education achieved (n = 641)		
Elementary school (1–8 years)	16	2.5
High school (9–12 years)	328	51.2
College/vocational school (13–16 years)	261	40.7
Professional/graduate (17–20 years)	36	5.6
Are you currently enrolled or participating in any school or training programs? (n = 632)		
Yes	99	15.7
No	533	84.3
Are you currently employed? (n = 529)		
Yes, part-time or full time	239	45.2
No	290	54.8
What is your current living situation? (n = 646)		
I own a home	45	7.0
I rent an apartment	379	58.7
I rent from public housing	114	17.6
I live in a shelter/I am homeless	34	5.2
I live with friends of family	74	11.5
Would you say in your neighborhood you are: (n = 435)		
Always afraid	21	4.8
Often afraid	43	9.9
Sometimes afraid	179	41.1
Never afraid	192	44.1
If yes (woman has insurance), what coverage do you have? (n = 557)		
Private insurance	69	12.4
Husky A (medicaid)	448	80.4
Other	40	7.2
Have you received treatment for stress, sadness/depression or anxiety? (n = 630)		
Yes	217	34.4
No	413	65.6
CESD score (n = 648)		
≥16	373	57.6
<16	275	42.4

#### Parenting Women’s Inability to Meet Their Family’s Basic Needs

Information related to the basic needs of parenting women in the sample (N = 670) can be found in Table 2. Issues with food insecurity were common, with 48.6% (n = 301) of women reporting that their food had run out before the end of the month and 68.7% (n = 437) worrying they would run out of food before they could buy more. Additionally, providing healthy food was a challenge—55.2% (n = 350) of women reported they often or sometimes could not purchase healthy meals and 40.5% (n = 250) reported not being able to provide healthy food to their children due to financial constraints. Women often sought public assistance to help alleviate issues with food insecurity—82.0% (n = 533) of women reported the receipt of food stamps and 70.9% (n = 410) participated in the Women, Infants, and Children (WIC) program. Additionally, 52.6% (n = 275) of women reported having lots of trouble or some trouble obtaining basic baby and child-related needs and supplies, including formula, food, clothes, and shoes. Finally, 50.3% (n = 300) reported the receipt of TANF.

**Table 2:** Basic needs of parenting women (N = 670)

Characteristic	n	%
Does your family ever worry about not having heat or utilities? (n = 638)		
Yes	300	47.0
No	338	53.0
Does your family ever run out of food before the end of the month? (n = 619)		
Yes	301	48.6
No	318	51.4
Are you currently or have you ever received food stamps? (n = 650)		
Yes	533	82.0
No	117	18.0
Are you now or were you ever enrolled in the WIC program? (n = 578)		
Yes	410	70.9
No	168	29.1
Are you now or have you ever received TANF? (n = 597)		
Yes	300	50.3
No	297	49.7
I worried whether our food would run out before I got money to buy more (n = 636)		
Often or sometimes true	437	68.7
Never true	199	31.3
I couldn’t afford healthy meals (n = 634)		
Often or sometimes true	350	55.2
Never true	284	44.8
I couldn’t feed my child/children healthy food because I couldn’t afford that (n = 617)		
Often or sometimes true	250	40.5
Never true	367	59.5
How much trouble do you have with paying for the following?		
Diapers for your child (n = 181)		
Lots of trouble or some trouble	100	55.2
No trouble at all or not applicable	81	44.8
Supplies like formula, food, clothes, and shoes (n = 523)		
Lots of trouble or some trouble	275	52.6
No trouble at all or not applicable	248	47.4
Other cleaning/hygiene supplies, such as shampoo, toothpaste, pads, tampons (n = 539)		
Lots of trouble or some trouble	261	48.4
No trouble at all or not applicable	278	51.6
If you have children in diapers, do you ever feel that you do not have enough diapers to change them as often as you would like? (n = 180)		
Yes	66	36.7
No	71	39.4
Not applicable	43	23.9

In a sub-sample of the population (N = 202), limited to parenting women with children under four (those with potential diaper need), 55.2% (n = 100) reported having lots of trouble or some trouble paying for diapers, and 36.7% (n = 66) reported not having enough diapers to change their child whenever they liked.

### Terminology Frequently Used by Parenting Women in Relation to Poverty

#### Overview of Results

Sources of stress related to poverty among parenting women were divided into four major themes: financial hardship, housing, employment, and difficulties with transportation. Several words and phrases were used frequently by parenting women in relation to these themes; frequencies of specific terms can be found in Table 3. It is important to note, that while these themes are presented as distinct categories, there was overlap between themes. For example, the financial situation of parenting women includes difficulty with bills, and in particular paying for rent, a theme also closely tied to housing instability. Furthermore, issues with employment were often related to an individual’s inability to pay their bills and afford adequate housing. Thus, while themes are presented as distinct, in many cases they overlap to create a picture of the synergistic nature of the stress experienced by parenting women in poverty.

**Table 3:** Frequency of specific words and phrases used by parenting women in relation to current stressors (N = 436)

Word or phrase	n	% of total responses
Financial hardship		
“Bill” or “bills”	45	10.3
“Money”	45	10.3
“Financial,” “financially” or “finances”	31	7.1
“Food” or “food stamps”	12	2.8
Employment		
“Job,” “jobs” or “jobless”	93	21.3
No job—“not having a job,” “not having job,” “no job,” “I don’t have a job,” “jobless” or “I lost my job”	38	8.7
Looking for job—“finding a job,” “finding and holding a job,” “looking for a job,” “looking for a different job,” “looking for a new job,” “job searching” “trying to find a job” or “trying to get a job”	14	3.2
“Work” or “working”	43	9.9
No work—“lack of work,” “not working” or “not being able to work”	5	1.1
“Looking for work”	1	0.2
“Employment,” “unemployment” or “unemployed”	14	3.2
No employment—“unemployment,” “unemployed,” “not having employment,” or “employment—lacks”	8	1.8
Looking for employment—“finding employment,” “looking for fulltime employment” or “finding permanent employment”	4	0.9
“Income,” “wage,” “wages” or “paid”	8	1.8
Housing		
“Housing,” “house,” “household,” “home” or “apartment”	35	8.0
“Living space,” “living situation” or “apartment situation”	12	2.8
“Rent,” “rental assistance” or “housing assistance”	10	2.3
“Homeless” or “homelessness”	9	2.1
“Safe,” “safety” or “unsafe”	6	1.4
Transportation		
“Car” or “bus”	10	2.3
“Transportation”	7	1.6
Parenting		
“Provide” or “providing”	5	1.1

#### Financial Hardship

Women frequently expressed concern over their current financial situation. Most commonly, women expressed difficulty paying their bills and difficulties with money, with the women using the terms “bill,” “bills” or “money” when describing sources of stress. A number of terms were used to describe difficulties with finances generally, such as “financial,” “financially” or “finances.” Finally, many of these financial difficulties resulted in difficulties with basic need obtainment, with women specifically mentioning “food” or “food stamps” as a source of stress.

#### Employment

Many of the financial hardships described above resulted from challenges that women experienced with employment. “Job,” “jobs” or “jobless” were the most frequently reported terms used by women in relation to stress, and were found in 21.3% (n = 93) of responses. Second most frequently, women described stress in relation to “work” or “working.” Additionally, the terms “employment,” “unemployment” or “unemployed” were used frequently by women. In many instances, women used terms related to jobs, work and employment in relation to their current unemployment, with phrases such as “not having a job” or “no job” being used most frequently. Finally, due to challenges with employment, many women expressed stress related to their pay. Specifically, “income,” “wage,” “wages” or “paid” were mentioned by women when discussing their current sources of stress.

#### Housing

Housing was also a common concern presented by parenting women. The most common terms used among parenting women were “housing,” “house,” “household,” “home” or their “apartment.” Additionally, women specifically listed difficulties with their “living space,” “living situation” or “apartment situation,” as problematic. For those in housing, rent often presented a difficulty, with women describing “rent,” “rental assistance” or “housing assistance” as a challenge. Safety was also a concern among women, with several experiencing robberies and homicides within close proximity to their neighborhood or housing. Specifically, women used the terms “safe,” “safety” or “unsafe.” Finally, a number of parenting women had difficulty obtaining housing and used the terms “homeless” and “homelessness” in relation to these difficulties.

#### Transportation

Lastly, several of the issues listed above were exacerbated by difficulties with transportation, as women had difficulty traveling to places they needed to go, including jobs. Specifically, women used the terms “car,” “bus” or “transportation” to describe these challenges.

### Qualitative Investigation of the Role of Poverty in Parenting Practices

#### Overview of Results

Many women described poverty in relation to their children and their parenting practices. Most frequently, this took the form of stress related to a woman feeling that she was unable to provide for her child or children in the way she wanted to. Some women also reported they felt they were missing important experiences and time with their children due to time constraints related to their financial situation. Finally, women reported custody issues related to their experience of homelessness.

#### Providing for Children

Financial difficulties put a particular strain on women, as many reported stress related to an inability to provide for their children and family. Women expressed the desire to do more for their children and to make sure all of their children’s needs were met, but were unable to do so given their current financial situation. The following are examples of how women described this struggle:

Being unemployed, not being able to provide for my family in a way that satisfies me…

…feel unable to provide children with friends, experiences

Being a single mom with three sons and not being able to give them everything that they need and want

Having better job, do more for my children (place to go, affordable)

Just need a new job and to make sure all my bills are paid and kids have what they need

Not having the total means to maintain and support myself and my family in a great way

#### Missing Out on Experiences

Several women described stress related to feeling that they were missing out on experiences or seeing their children due to their need to work to provide for their household. This was a phenomenon that was noted as especially stressful for single mothers. The following are examples of how women described this source of stress:

…working so much and feeling like I’m missing out on my son to make ends meet

Working full time; going to school and raising 3 children as a single parent…

#### Homelessness

While some women discussed the experience of homelessness generally, many discussed homelessness in relation to their children. In some cases, women were able to maintain custody of their children whilst homeless, while in others they reported the loss of custody of their children or the separation from their family as a result of homelessness:

Homelessness, lost custody of my children

Homeless with 2 year old son. DV victim, recurrent flashbacks of abuse, not enough money for food diapers or clothes

Being homeless. My husband and kids aren’t living with me. Our family being displaced

## Discussion

### Overview

This research provides a base for understanding poverty specific to low-income parenting women in several ways. We were able to quantitatively operationalize poverty specific to a sample of low-income mothers, examine how mothers describe sources of stress related to poverty, and explore how the experience of poverty affects women’s parenting practices.

### Operationalizing the Experience of Poverty Among Parenting Women

First, we found that parenting women described the supports that were critical to helping them meet their basic needs. Among urban, African American and Hispanic mothers living in high poverty, we found that the most critical supports women described were SNAP (food stamps), WIC, and TANF.

Additionally, in spite of the fact that such a high percentage of our women received benefits such as SNAP and TANF, many women still experienced difficulty obtaining basic needs related to parenting, such as food, diapers, and clothing. Difficultly obtaining basic needs is a risk factor for chronic mental health challenges for both mother and child. For example, food insecurity has been associated with an increased likelihood of mental health conditions among mothers, with those described as being food insecure having a prevalence of generalized anxiety disorder or a major depressive episode 13.4% higher than those described as fully food secure (Whitaker et al. 2006). Furthermore, women who report the need for mental health services are more likely to report the need for diapers (Smith et al. 2013). Difficulty meeting basic needs has also been associated with a number of adverse child outcomes. For example food insecurity is related to increased externalizing and internalizing symptoms in children (Slopen et al. 2010).

The difficulty that low-income parenting women face obtaining basic needs and the frequency with which low-income parenting women are also connected to healthcare providers through pediatric well child visits, prenatal and postpartum care, and through schools or early childcare settings, presents a potential opportunity for healthcare providers, educators and child caregivers to screen for unmet basic needs and to ensure institutions (healthcare, education and early childhood care) are connected to community agencies providing basic needs supports. For example, these screenings could occur in the context of Head Start programs, which could be doubly beneficial, given that these programs are associated with an increase in educational attainment for some parents (Sabol and Chase-Lansdale 2015). We suggest an additional method whereby this might occur.

A Basic Needs-Informed Curriculum, has been developed by the National Diaper Bank Network and the MOMS Partnership to train professionals on the importance of addressing basic needs for families (Goldblum and Smith 2015). One aim of this program is that healthcare and social service professionals will have the capacity to have conversations with low-income individuals about basic needs and form connections with community agencies to partner around increasing a family’s ability to meet their basic needs (Goldblum and Smith 2015). Healthcare providers, early childcare workers and social service employees could all benefit from the basic needs curriculum. Over 330 individuals have been trained to date on this curriculum (Smith et al.). Early results suggest this curriculum is effective in helping individuals understand basic needs, including how they can affect families and stress levels, and in helping individuals understand the way they or their organization can support individuals experiencing difficulty obtaining basic needs (Smith et al.).

### Terminology Frequently Used by Parenting Women in Relation to Poverty

Second, we describe how mothers talk about issues of poverty and basic needs using the terms they use as compared to terms that may be frequently used by social service and healthcare providers. We found mothers frequently talked about “bills,” “money,” “jobs,” “work,” “housing” and “transportation,” rather than talking about “poverty,” “at risk” or “low-income,” words commonly used by researchers and practitioners. Given the importance of these terms in describing stress, it is critical that healthcare providers incorporate these terms into clinical assessments with low-income, parenting women. Matching clinical language with terms that patients and clients utilize themselves has been found to improve patient satisfaction (Williams and Ogden 2004), which could increase the likelihood that individuals will continue with treatment and follow medical advice. It is especially important that the terminology and experiences of low-income women are incorporated into training curriculums for healthcare providers, as recent research has demonstrated that medical residents have limited knowledge of underserved populations, as well as limited confidence in their knowledge (Wieland et al. 2010). Further, fewer than 15% of students at one medical school had knowledge of programs for low-income individuals, such as WIC (Doran et al. 2008).

### Qualitative Investigation of the Role of Poverty in Parenting Practices

Third, women described how poverty directly negatively impacted their ability to provide the quality of parenting they wanted to achieve, primarily through restricting the amount of time they could spend with their children and in limiting their ability to provide experiences to their children. This finding is in keeping with past literature which has shown that parental monitoring may be eroded in the contexts of high poverty (Costello et al. 2003). Further, research has shown that social capital is critical for child development and outcomes (De Silva et al. 2005; Furstenberg Jr and Hughes 1995; Parcel and Menaghan 1993). However, this might be restricted among low-income parenting women and families, unless these individuals are tied to community institutions that can serve as resource brokers, defined as “organizations that have ties to businesses, nonprofits, and government agencies rich in resources and that provide their patrons with access to these resources” (Small 2006). The MOMS Partnership currently serves as a resource broker for low-income, parenting women.

### Limitations

Several limitations must be considered when interpreting the results of this study. First, study participants were selected from a single, urban city. Thus, results may not be generalizable to all parenting women living in poverty, such as those living in rural areas. For example, while the terms identified in our study provide a start for providers who serve low-income, parenting women in an urban setting, they are likely not generalizable given our sample. Second, all survey information was obtained via self-report, and thus we are not able to ascertain if women completely comprehended all of the questions. While we attempted to minimize this concern by allowing women to skip questions, this resulted in a higher percentage of missing data for certain questions, although we did not observe systematic patterns of missing responses.

### Future Research

Future research should further expand on the operationalization of poverty amongst parenting women. Specifically, research is needed in diverse regions of the United States, such as rural settings, and in partnership with low-income parents themselves to increase effectiveness of research design and community benefit that accounts for the context of poverty and the mechanisms by which material hardship specifically impacts parenting outcomes. Additionally, further qualitative research is necessary to examine how parenting practices are affected by the experiences of poverty among racial and ethnic subgroups, as this research has high potential for tailoring interventions. Lastly, the development of curriculums for healthcare providers using language frequently used by low-income women should be considered and efficacy of such a curriculum should be established.
